# Editorial: Neural immuno-inflammatory response in neurodegenerative diseases

**DOI:** 10.3389/fneur.2023.1133757

**Published:** 2023-01-23

**Authors:** Xintong Ge

**Affiliations:** ^1^Haihe Laboratory of Cell Ecosystem, Department of Geriatrics, Tianjin Medical University General Hospital, Tianjin, China; ^2^Tianjin Geriatrics Institute, Tianjin, China

**Keywords:** immunity, inflammation, neurodegeneration, Alzheimer's disease, traumatic brain injury, chronic traumatic encephalopathy, stroke, vascular dementia

Neurodegeneration from Alzheimer's disease (AD), Parkinson's disease (PD), stroke, neurotrauma, and other brain diseases could lead to dementia and severe cognitive disorders. In all these conditions, the cellular and molecular mechanisms underlying the occurrence and development of neurodegeneration are expected to be fully elucidated. These mechanisms include immune regulation, chronic inflammatory response, production and deposition of pathological proteins, blood-brain barrier damage, etc. (1–5). Although this research field has attracted much attention in recent years, there is still a lack of in-depth mechanistic study in most fields, which may limit the exploitation of novel therapeutic strategies in the future. This Research Topic aims to shed more clarification on the regulation mechanisms of neural immuno-inflammatory response that leads to neurodegeneration. Herein, four articles provide new insights into the pathogenesis and potential treatments for neuroimmune dysfunction and neuro-inflammation in neurodegenerative diseases.

The first article (Santoro et al.), *Assessment and Diagnosis of Down Syndrome Regression Disorder: International Expert Consensus*, is strongly recommended by the editors. Down Syndrome Regression Disorder is a debilitating neurocognitive disorder. Although phenotyped, no diagnostic criteria exist for this condition. Lack of standardized assessment tools has slowed research in this clinical area. The authors performed a two-round traditional Delphi method survey of an international group of clinicians with experience in treating Down syndrome to develop a standardized approach to clinical care and research. An international consensus agreement on the nomenclature, diagnostic work up and diagnostic criteria for Down Syndrome Regression Disorder was finally presented, which provides an initial practical framework that can advance both research and clinical practices for this condition.

The second review article (Chen et al.) focused on the lymphatic system in the central nervous system (CNS), which is closely associated with neural immunity and transportation of pathological proteins. Specifically, the presence of lymphatic vessels lined with endothelial cells in the meninges has been widely confirmed in recent years. The periventricular meninges host different populations of immune cells that affect neural immune response, and the continuous drainage of interstitial and cerebrospinal fluid also proceeds mainly by the lymphatic system. On this basis, this review further discussed latest advances in intracranial lymphatic circulation and the pathogenesis of its associated diseases, including infectious diseases, autoimmunity, and tumor immunology. Novel therapeutic strategies for the diseases associated with the intracranial lymphatic system were also summarized.

The third research article (Duan et al.) identified a group of genetic molecular markers of AD, including ATP2A2, ATP6V1D, CAP2, and SYNJ1 through weighted gene co-expression network analysis (WGCNA). Gene Ontology and pathway enrichment analysis suggested that these hub genes of the network play significant roles in the differentiation and growth of neural cells and transmission of neurotransmitters. Gene set enrichment analysis for the genes showed a significant enrichment in immune/infection pathways. In addition, an investigation on immune infiltration characteristics revealed that activated mast cells, regulatory T cells, plasma cells, neutrophils, T follicular helper cells, CD8 T cells, resting memory CD4 T cells, and M1 macrophages were the core immune cells contributing to AD progression. These findings provide novel potential biomarkers for AD, and further indicate the importance of immune pathways and immune cells in the occurrence and development of the disease.

The fourth article (He et al.) is a meta-analysis, which studies the relationship between inflammatory rheumatic diseases and the occurrence risk of PD. It indicates that ankylosing spondylitis, Sjögren's syndrome and Behcet disease are correlated with an increased PD risk, whereas no associations were observed between gout, rheumatoid arthritis, systemic lupus erythematosus as well as polymyalgia rheumatica and the subsequent development of PD. The research contributes to promoting active screening and prompt treatment of Parkinson's Disease from the perspective of immune-inflammatory response.

In the last mini review (Deng et al.), the authors highlighted the significance of N6-methyladenosine (m6A), the most prevalent post-transcriptional RNA modification, in CNS functioning and development. Emphasis was placed on recent findings that elucidate the molecular functional mechanism of m6A modification after brain injury and cerebrovascular disorders. A neurobiological basis for further investigation of potential treatments was also described.

While the five articles have broadened our previous understanding on the roles of neural immuno-inflammatory response and its regulatory mechanism in various neurodegenerative diseases, its associated intervention strategies still remain to be studied. Further research from *in-vitro* to *in-vivo* models is needed to be designed using novel bio-techniques, such as single cell sequencing and spatial transcriptomics, aiming at exploring effective treatments for clinical application.

## Author contributions

XG wrote the manuscript, was one of the editors of this Research Topic, and selected the articles described herein. All editors agreed to authorize XG to write this editorial on behalf of the guest editorial team, and approved the submitted version.
